# ‘Survivorship care is one big gap’: a qualitative study of post-treatment supportive care in Aotearoa New Zealand

**DOI:** 10.1186/s12913-023-09580-8

**Published:** 2023-06-08

**Authors:** Jerram Bateman, Richard Egan, Karyn Maclennan

**Affiliations:** 1 Social and Behavioural Research Unit, Department of Preventive and Social Medicine, University of Otago, PO Box 56, Dunedin, 9054 New Zealand; 2 Ngāi Tahu Māori Health Research Unit, Division of Health Sciences, University of Otago, PO Box 56, Dunedin, 9054 New Zealand

**Keywords:** Survivorship, Post-treatment care, Supportive care, Service providers

## Abstract

**Background:**

This study focuses on the provision of supportive care services and programmes for cancer survivors post-treatment in Aotearoa New Zealand (NZ). It aims to aid our understanding of an often challenging and fragmented phase of cancer survivorship, and lay the groundwork for future research into the development of survivorship care in NZ.

**Methods:**

This study employed a qualitative design using semi-structured interviews with a range of healthcare providers (*n* = 47) involved in service provision for cancer survivors post active treatment, including supportive care providers; clinical and allied health providers; primary health providers; and Māori health providers. Data were analysed using thematic analysis.

**Results:**

We found that cancer survivors in NZ face a range of psycho-social and physical issues post-treatment. The provision of supportive care to meet these needs is currently fragmented and inequitable. The key barriers to improved supportive care provision for cancer survivors post-treatment include a lack of capacity and resources within the existing cancer care framework; divergent attitudes to survivorship care within the cancer care workforce; and a lack of clarity around whose responsibility post-treatment survivorship care is.

**Conclusions:**

Post-treatment cancer survivorship should be established as a distinct phase of cancer care. Measures could include greater leadership in the survivorship space; the implementation of a survivorship model(s) of care; and the use of survivorship care plans; all of which could help improve referral pathways, and clarify clinical responsibility for post-treatment survivorship care.

## Background

In cancer, survivorship gives emphasis to the health and wellbeing of a person with cancer from the point of diagnosis, until the end of life, and includes specific assessments, programmes and services focused on living with, through and beyond cancer [1]. Within the context of improved cancer survival rates over the past 40 years, cancer survivorship has received increased attention in international research and policy [2]. In Aotearoa New Zealand (NZ), the government has recently responded to survivorship needs, pledging up to $4.2 million a year towards the Cancer Psychological and Social Support Initiative for adults with cancer [3]. In addition, an advisory group made up of a wide-range of stakeholders was set up to develop a consensus statement on cancer survivorship in NZ, to “provide a foundation to inform policy development, evaluate existing services and as a guide for establishing new initiatives and services” [1]. The consensus statement advisory group was predicated on the fact that there has been limited policy and research work undertaken on cancer survivorship in NZ, a sentiment O’Brien et al., who’s seminal study on the wide-ranging impacts reported by cancer survivors remains one of the few studies undertaken from a survivorship lens in NZ, agree with, stating that there is limited survivorship literature with a NZ perspective, and consequently the evidence base on survivorship in NZ is inadequate [4]. It is therefore imperative that research seeking to better understand the needs of cancer survivors in NZ, and the extent to which current supportive care provision is addressing those needs, is undertaken in order to address this paucity of knowledge.

One issue emerging from the international survivorship literature is that, despite advances in cancer care, survivors still report unmet needs after completing active treatment [2, 5]. Burg et al. define unmet needs as “…needs which lack the level of service or support an individual perceives are necessary to achieve optimal wellbeing.” [6], and in the context of survivorship care, they include psychosocial; physical; spiritual; resource and informational needs [2]. Recommendations for survivorship care once treatment finishes include the use of survivorship care plans, treatment summaries, follow-up care plans, improved communication and coordination of care between specialists and primary care providers, and programmes that address physical, social, cultural, emotional, nutritional, informational, psychological, spiritual and practical aspects of care [5, 7–10]. For example, the Clinical Oncology Society of Australia recommends that all patients receive a care plan as part of their transition from the acute care setting back to the community [11], the National Health Service, UK supports the use of holistic needs assessments, care plans, and treatment summaries to mark the end of treatment [12], while the American Society of Clinical Oncology proposes a shared care plan that promotes the effective transition of patients from the oncology setting to a primary care setting after active treatment [8].

While these recommendations have been implemented, there is considerable variation in design and implementation [13, 14], and limited evidence of their efficacy, and/or transferability to other settings. Some studies have shown that cancer survivors and healthcare providers find supportive care plans beneficial to co-ordination of care; improved referral pathways; the delineation of roles and responsibilities; and provision of information on follow-up care [14–17]. Other studies have described barriers to the provision of survivorship care plans to cancer survivors, including lack of time and resources among healthcare providers; lack of clarity around who is responsible for preparing the plan and lack of reimbursement for this role; and poor communication and collaboration between providers [2, 16, 18, 19]. Further research is therefore needed to test different variations of survivorship care plans, and their efficacy and cultural safety, in a range of settings [17, 20, 21].

Similarly, many models of care for cancer survivorship have been researched and developed in the past decade, but there is little evidence of their efficacy [2, 22–24]. Shakeel et al. argue that “despite the widespread acknowledgment of the importance of integrated survivorship care within these models, the optimal survivorship care model that clarifies practitioner roles and responsibilities remains poorly understood, and implementation of efforts remains fragmented” [24]. It is generally accepted that there is currently no optimal model of care that will work for all cancer survivors [2, 23, 24], but a broad, holistic, integrated model that can be tailored to address individual needs, could help clarify practitioner roles and responsibilities, and ensure more equitable access to post-treatment survivorship care [24].

While there are now a number of survivorship-focused services being delivered in NZ, these initiatives have largely been focused on the “front of the treatment pathway” rather than post-active treatment [3]. The *New Zealand Cancer Plan 2015–2018* emphasised the role of survivorship care post active treatment, stating that “follow-up care, involving monitoring and surveillance as well as physical, psychological and social support to help people manage the short and long-term effects of their cancer, can improve a person’s quality of life” [25]. The recently released *New Zealand Cancer Action Plan 2019–2029* has built on this, outlining the need for person-specific care plans that meet the holistic long-term needs of cancer survivors [26]. Future directions and expectations for follow-up cancer care in NZ include the assessment of a person’s supportive care needs. This includes greater access to emotional, psychological, spiritual and social support, such as practitioner-led and patient-led support groups, counselling services, and return-to-work programmes. Further, it needs improved communication between people affected by cancer and their primary and secondary healthcare providers; consistent follow up from all clinicians; and the addressing of equity issues [26].

In line with the future directions and expectations for follow-up cancer care in NZ outlined above, and following the lead of the consensus statement advisory group, this study seeks to investigate cancer care delivery practices and the coordination of supportive care during the transition from active treatment to post-treatment, from the perspectives of a range of healthcare providers. This study focuses on the current provision of supportive care services and programmes for cancer survivors post-treatment in NZ. The study seeks to identify models of care, barriers to transferral of care, integration between service providers, referral pathways, and resources available for cancer survivors in the transition from active treatment to long-term follow-up care. It also examines service provider engagement with underserved populations, particularly Māori, in the transition from active treatment to long-term follow-up care. In doing so, it aims to aid our understanding of an often challenging and fragmented phase of cancer survivorship [2], and lay the groundwork for future research into the development of survivorship care in NZ.

## Methods

### Design

This study employed a qualitative design using semi-structured interviews with a range of healthcare providers involved in service provision for cancer survivors in the transition from active treatment to long-term follow-up care. It is therefore exploratory in nature, which is best suited to a qualitative approach [27].

### Recruitment

Participants for this study were recruited through a mixture of purposive and snowball sampling techniques. Purposive sampling selects information rich cases for in-depth study, from which the researcher can learn a great deal about the research focus [28], whereas snowball sampling is where those selected via purposive sampling are asked if they know of others who may be able to provide further insight on the themes of the research [29].

Potential participants were identified from health provider websites, and community, stakeholder and study investigators’ networks. Initial contact with these providers was made through an email introduction from the study investigators, inviting them to participate in the study. This email included detailed information about the nature and scope of the research, as well as their rights as a participant should they wish to participate. From there, a snowballing technique was utilized, whereby each participant was asked what other supportive care providers they knew of, refer to, or interact with, with relation to the provision of supportive care for cancer survivors in the transition from active treatment to long-term follow-up care.

### Data collection

We conducted in-depth semi-structured interviews with a range of healthcare providers involved in the treatment and supportive care of cancer survivors across NZ. An interview schedule was developed and refined after an extensive review of the literature, though as noted above, the interviews were semi-structured, so the precise questions varied depending on how the interview developed. Due to COVID-19 restrictions, all interviews were conducted either on the phone or via Zoom, and informed consent was obtained from the participant verbally at the beginning of the interview. Interviews lasted 30–78 min, and with the consent of participants, were audio-recorded.

### Data analysis

This research adopted a pragmatic methodological position, allowing for the voices of the participants to lead interpretation [30]. Audio recordings of the interviews were transcribed verbatim, transcripts were checked against the audio-recordings for accuracy and managed using NVivo software. The transcripts were analysed using thematic analysis, a fluid method which identifies, analyses and reports patterns within the data, and can easily be applied in different theoretical frameworks [31]. Analysis involved coding of repeated words and phrases; evaluating relationships between codes; identifying patterns, commonalities and differences; and creating a set of higher-order themes [31]. Emergent themes and subthemes were then analysed by the authors. Thematic analysis is a recursive process, and involved back and forth movement between each of these steps [28]. Data and discussions of each theme are integrated in the following section to provide a cohesive interpretation for readers.

### Participant characteristics

The sample (*n* = 47) included cancer supportive care providers (*n* = 19), such as supportive care coordinators, supportive care nurses, and managers, representing cross-tumour Cancer NGOs such as the Cancer Society and tumour specific organisations; clinical and allied healthcare providers (*n* = 13) such as oncologists, cancer nurse coordinators, clinical nurse specialists, physiotherapists and psychologists; primary health providers (*n* = 9) such as general practitioners, practice nurses, and health navigators; and Māori health providers (*n* = 6). The majority of participants worked in cross-tumour or general health services (*n* = 40), with the remaining seven participants involved in tumour-specific services, including breast, colorectal, Leukaemia and blood, prostate, and head and neck cancers.

In terms of geographical location, participants were spread relatively evenly throughout the country. In 2022, health reforms in New Zealand saw 20 District Health Boards (DHB) merged into Te Whatu Ora – The New Zealand Health Authority, which consists of 4 regional divisions—twelve participants lived within the Northern Region (upper North Island); 10 in Te Manawa Taki (Central North Island); 10 in Central (Lower North Island); and 15 in Te Waipounamu (South Island). At least one participant was drawn from within each of the former DHB boundaries, further highlighting geographical spread.

Participants were almost exclusively female (*n* = 45), and predominantly Pākehā (NZ European) (*n* = 39) and Māori (*n* = 8).

## Results

Four overarching themes were generated from the interviews with healthcare providers relating to cancer survivors post-treatment: 1. Key issues cancer survivors face post-treatment; 2. Current supportive care provision for cancer survivors post-treatment; 3. Barriers to supportive care provision for cancer survivors post-treatment; and 4. Equity within supportive care provision for cancer survivors post-treatment. Within each of these overarching themes, numerous sub-themes were identified and will be outlined in the following subsections, illustrated with exemplar quotes.

### Key issues cancer survivors face post-treatment from the perspective of healthcare providers

Cancer survivors face a wide variation of issues post-treatment, influenced by factors such as type of cancer, type of treatment, age, gender, ethnicity, and employment. The cancer journey, or “roller-coaster” as one participant described it, is different for all survivors, resulting in different needs as they transition from active treatment to long-term follow-up care. That said, a number of common issues facing cancer survivors post-treatment were evident from the interviews, particularly in relation to their psychological and emotional needs, but also their physical, and social needs.

#### Sense of abandonment

Participants commonly referred to a sense of abandonment as being one of the key issues cancer survivors grapple with post-treatment. Cancer survivors were described as being ‘wrapped around’ by health professionals and supportive care providers from high-suspicion of cancer through to the end of treatment. Once active treatment is completed, however, contact with health professionals and supportive care providers typically becomes much less frequent. While participants largely agreed that this is acceptable from a clinical perspective, they acknowledged that the reduction in contact post-treatment often leads to feelings of abandonment and anxiety among cancer survivors, as Participant 37 (Supportive Care Provider) describes:

“they have a lot of ‘wrap around’, and then they get discharged, and that social connection is lost. Some people even talk about how they really miss going to the hospital for treatment, because they really miss that social connection, and the comfort they experience with it. So it’s mainly around feeling lost, or abandoned, you know, how do I look after my health moving forward? What’s the plan? Who’s going to help me look after my health moving forward? Am I going to be reviewed? And these feelings can lead to complex issues around anxiety and depression.”

It was also noted by many participants that the support of family and friends often begins to subside during the transition from active-treatment to follow-up care, as there is a tacit expectation that life ‘returns to normal’:

“they [survivors] get to the end of treatment, and family and friends are celebrating, and ‘isn’t it great, you got through this’. In terms of support, relationships often go back to how they were pre-cancer. Most people don’t understand that life has changed, they just think you are clear of cancer, get on with it” (Participant 19, Supportive Care Provider).

#### Fear of recurrence

A specific anxiety stemming from the sense of abandonment outlined above, was described by participants as a ‘fear of recurrence’. They noted that while in treatment, cancer survivors often find some comfort in the regular monitoring and contact from health professionals described above. But there is a fear that changes to their health may be missed once monitoring becomes less frequent post-treatment. This point is highlighted by Participant 16 (Supportive Care Provider), who states:

“Once you’ve had a cancer diagnosis, it never actually leaves you, it’s always in the back of your mind. That fear of recurrence and information around that, is something that I think most people struggle with post-treatment, I would say it’s universal. It’s not that well-addressed during treatment and is something people are left with – I mean they’re given the stats, at 5 years blah, blah, but how do you negotiate that information about a way of actually getting through that fear of recurrence…you know, what do I do if I suddenly have a pain here? I know when I was in treatment, I could ring the ward, or whatever. Whereas now it’s like, oh, I’ve got a pain here, who do I talk to about that? What do I do if I’m worried about something? And often it’s, I’m going to have a scan in 6 months, but what happens if it’s growing now? Right now?”

Somewhat paradoxically, participants also described a heightened fear of recurrence in the context of follow-up surveillance as being a major source of anxiety for cancer survivors post-treatment. A number of participants used the term ‘scanxiety’ to embody feelings of anxiety and fear felt by cancer survivors leading up to, and awaiting the results of, scans and medical tests conducted routinely post-treatment:

“When you have to go back and have another scan, and you know, if you check every year, are you going to find out that this is the year it comes back? You talk to anyone that goes through that, it makes people incredibly anxious…there’s very little support for that.” (Participant 20, Supportive Care Provider)

Thus, fear of recurrence was not only considered a consequence of reduced contact with health professionals, but also potentially triggered by contact with health professionals, post-treatment.

#### Emotional and psychological processing

A number of participants talked about post-treatment being the first opportunity for many cancer survivors to process the emotional and psychological toll cancer had on them. As Participant 37 describes, survivors are often so focused on getting through the treatment, that they do not have the capacity to process the emotional and psychological impacts of a cancer diagnosis:

“often people are just on autopilot, hunkering down to get through the diagnosis and get the treatment done, and its not until they finish treatment and are out the other side, that they have an opportunity to process it, and it all comes crashing in on an emotional level.” (Participant 37, Supportive Care Provider)

This emotional and psychological processing post-treatment was described by participants as a ‘grieving process’ and ‘existential crisis’, where cancer survivors are faced with the fact that their life had changed irrevocably:

“It’s often working through the grief process of what has happened, because it almost seems that when going through treatment, the focus is on getting to the end of treatment. And that’s where it is ‘I’m going to beat this’, ‘I’m going to live’, then they get there and all of a sudden, the reality of what life looks like post-treatment hits them. And it’s a very different place to what life was like pre-diagnosis” (Participant 12, Supportive Care Provider).

Participants also suggested that the anxiety induced by this period of emotional and psychological processing is heightened because it tends to occur in conjunction with the ‘sense of abandonment’ outlined above:

“The physical part of treatment, be it chemo, or radiation, or whatever, is just so physically demanding that the emotional processing hasn’t had time to catch up. And it’s when they’ve got to the end, and all the support has disappeared, because they’re not seeing, you know, their doctor regularly, they’re not back every 3 weeks for chemo, they’re not having radiation. So those relationships that they’ve built up during treatment suddenly disappear at about the point that their emotional processing of what’s actually going on starts.” (Participant 16, Supportive Care Provider)

#### Sense of abnormality

A sense of abnormality, driven by both internal and external expectations, was also raised by participants as a common issue facing cancer survivors post-treatment. Feelings associated with the sense of abandonment, fear of recurrence, and emotional and psychological processing, described above, are often internalised as abnormal by cancer survivors who have finished treatment, a point exemplified by Participant 13 (Supportive Care Provider):

“I hear it all the time – ‘I’m living, and I should be grateful, but I just want to cry. I’m anxious, I’m overwhelmed and really emotional. What’s wrong with me?”

These feelings of abnormality are often amplified by external expectations, and can be a significant barrier to cancer survivors seeking support post-treatment, as Participant 36 articulates:

“[Survivors] will get an offhand comment from somebody about, often just one comment, you know, why aren’t you back at work? Why do you need an afternoon nap? And they start feeling really embarrassed and conscious of why, and think something is wrong with them. They are often reluctant to seek help because they feel ungrateful for bringing those kinds of things up when there are people who are going through cancer and still have that uncertainty about what the final outcomes are going to be. It’s not until they get into a support group setting with other survivors, where it’s [post-treatment issues] actually talked about, that they realise they’re actually quite, for want of a better word, normal, and that society’s expectation on them is unrealistic.” (Participant 36, Supportive Care Provider).

#### Physical issues

There were no clear sub-themes around the specific physical needs of cancer survivors post-treatment, possibly because the physical impact of cancer varies so widely between different cancer types, and different treatment types. However, there was a general consensus among participants that all cancer survivors face some form of physical rehabilitation following treatment for cancer, as participant 15 neatly articulates:

“The physical part of treatment, be it chemo or radiation or surgery or whatever, is just so physically demanding. Be it losing a body part, or losing general strength, most people end up on this low, physiologically, post-treatment, that requires time and rehabilitation before they even vaguely feel normal again.” (Participant 15, Supportive Care Provider).

#### Social issues

Participants largely referred to social issues faced by cancer survivors post-treatment in relatively general terms, for example “…and there are a number of social issues survivors face following treatment” (Participant 40, Primary Health Provider). But some specific commonalities were evident, most notably returning to work:

“There can be high expectations on the person to get back to work ASAP, within themselves and from their employer, but the actual reality of returning to work often hits them like a tonne of bricks” (Participant 36, Supportive Care Provider)

Other social issues raised by participants included dealing with relationship problems that emerge, or compound, during the cancer journey; and financial concerns caused by loss of income, cost of treatment, or expenses relating to accessing treatment (travel, accommodation etc.).

### Current supportive care landscape for cancer survivors post-treatment

#### Patient-centred, but fragmented

As noted above, cancer survivors face a wide variation of issues post-treatment, resulting in a wide range of needs as they transition from active treatment to long-term follow-up care. Consequently, where it is accessible, current post-treatment supportive care provision is guided by the individual needs of each survivor, with participants across the range of supportive care provision interviewed typically describing their role with post-treatment survivors as ‘meeting them where they are at’, ‘problem solving’, or ‘finding solutions’:

“It’s very client centred. What they bring is what we work on” (Participant 14, Supportive Care Provider).

This commonly involves identifying issues the survivor and/or their whānau require support with, and either providing that support themselves, or identifying services within their organisation, the wider health system, or the community, that can provide the necessary support:

“I nurse the system as much as I nurse the patient, because it’s that care coordination and problem solving when there’s complexity in the disease, but there’s also complexity in the person or their social context that requires different solutions to the standard.” (Participant 7, Clinical and Allied Health)

While the above quote highlights the value in the patient-centred nature of this approach, participants also commonly referred to the current post-treatment supportive care landscape as ‘fragmented’, ‘disjointed’, and ‘ad hoc’, as a consequence of it:

“It’s [post-treatment supportive care] pretty disjointed. We sort of make it up as we go along, based on clinical experience, and patient needs at the time.” (Participant 1, Clinical and Allied Health).

Many also acknowledged that it is patient-driven, placing the onus on cancer survivors to seek supportive care at the end of treatment, which can result in unmet needs post-treatment:

“There are patients that I pick up who are a long way down their survivorship journey, with all sorts of problems, and you ask them if they’ve been referred to, you know, whatever support care service, and no-one has ever offered them that. They don’t know what they don’t know” (Participant 9, Clinical and Allied Health).

#### Provisions

Participants described a range of services that they either offer themselves, or refer cancer survivors to, to meet their post-treatment needs, including psychological, counselling and social work services; DHB support services; Cancer NGOs; and community organisations. Support provided was generally in the form of one-on-one sessions, support or exercise groups, home visits, webinars, and written resources. Innovation in peer-to-peer support through online platforms such as Support Crew [32] and Ripple [33], was also acknowledged by some participants.

While overall a wide range of services were discussed, there was little consistency between participants in terms of specific organisations that they would refer patients to for post-treatment supportive care. The Cancer Society and ‘Pinc and Steel’ (an initiative focused on physiotherapy for cancer survivors), were the only two organisations that were mentioned frequently by participants, when discussing the organisations they refer cancer survivors to for post-treatment care:

“We don’t have a lot of places to refer people to. We use the Cancer Society a lot.” (Participant 6, Clinical and Allied Health)

“the main one is the Cancer Society, because everybody knows who they are, and what they do.” (Participant 11, Clinical and Allied Health).

“Pinc and Steel physiotherapists are kind of scattered all over New Zealand, the world actually, it’s international. And anyone from diagnosis to 10 years post-diagnosis can access funding, so it’s a really great network for people to access.” (Participant 34, Clinical and Allied Health)

#### Gaps

Broadly speaking, there was an underlying sentiment that post-treatment supportive care was a gap, in and of itself, within the gamut of cancer treatment and supportive care, as Participant 15 (Supportive Care Provider) so succinctly surmised:

“Survivorship care is one big gap.”

This sentiment, while extreme, aligns with comments of others which indicate few services, if any, exclusively target the post-treatment needs of cancer survivors. Participants generally work with cancer survivors across the cancer continuum, but the front of pathway tends to be prioritised:

“I think we could do some more work around the survivorship specific support groups, and webinars and education and stuff. I think a lot of it is disease specific and treatment specific, but I don’t think we do as much around post-treatment, sort of, survivorship stuff.” (Participant 19, Supportive Care Provider).

Consequently, the post-treatment supportive care needs of cancer survivors tend to be met within the existing framework of supportive care services, rather than services and programmes specifically designed to meet their needs:

“I guess the thing about survivorship, if you bring those post-treatment people together, as a group, they then become support for each other, and can build each other’s skillset and resilience. That’s probably you know, what we’re lacking. We will have pockets of that in our support groups, but we have nothing established specifically for post-treatment.” (Participant 20, Supportive Care Provider).

More specifically, participants perceived psychological and emotional support to be a major gap in service provision, which aligns with their identification of psychological and emotional wellbeing as a key issue for cancer survivors post-treatment, as outlined above. Participants also flagged sexuality and fertility services; budgeting and financial services; and dietary and nutrition services, as significant gaps in the current post-treatment supportive care landscape.

“We really would love exercise physiologists, we’d love a dietitian. We’d love more psychological support to send people to you know, we’d love budgeting advice, social workers to talk about financial problems.” (Participant 1, Clinical and Allied Health).

“sexual dysfunction [is a gap], we do that really, really poorly. And I think it’s skipped a lot of the time. And I think there’s various reasons because there’s nothing available. So what can you do? And then, you know, the classic, it’s just a bit awkward, or making some assumptions that maybe that persons no longer sexually active.” (Participant 5, Clinical and Allied Health).

### Barriers to supportive care provision for cancer survivors post-treatment

#### No ownership of the post-treatment survivorship space

One of the broad reasons articulated for the fragmented nature of post-treatment supportive care was that no single body or organisation has taken ownership of the survivorship space. This point is clearly explicated by Participant 28 (Supportive Care Provider) who states:

“I think that there’s a real gap in terms of no-one actually claiming that survivorship space…so in the post-treatment phase, there is a real focus on the clinical follow-up, but it’s really, really clear that there is a huge gap in meeting the psychosocial needs, and that’s just not mentioned enough and not talked about, and no-one has really taken ownership of that space.”

Consequently, a range of different organisations and roles currently contribute to post-treatment supportive care, without any overarching framework or leadership to guide them, as Participant 7 (Clinical and Allied Health) illustrates:

“It isn’t clear who is ultimately responsible for post-treatment support. And I think as we move more and more to pushing longer term follow-up care back into the community and primary care, and emphasise self-management, this becomes more challenging for people.”

#### No clear referral pathways

Stemming from the lack of ownership of the survivorship space outlined above, many participants suggested that there were no clear referral pathways from front of pathway to post-treatment supportive care:

“There aren’t really the kind of outpatients’ services, with the knowledge of other services, or the ease of access to other services, that would be really helpful. There’s no pathway where we say, right, you have finished treatment, we will continue to monitor the cancer through ongoing surveillance, but all your other needs will be managed by these guys over here” (Participant 8, Clinical and Allied Health).

Some participants indicated this lack of clarity around referral pathways can lead to a reluctance to ask questions around cancer survivors’ post-treatment needs:

“There are lots of services that can be drawn on, but it depends largely whether the person referring knows about those services. Sometimes clinicians don’t ask [patients] because they don’t know what the solution to what’s presented to them might be.” (Participant 7, Clinical and Allied Health)

Participants also often acknowledged that they would not generally follow-up on referrals that they made, which can lead to cancer survivors falling through the cracks once they have finished treatment:

“Because of capacity, unless you get a bounce back saying the referral is declined, you probably wouldn’t follow-up…which is a place that gaps can occur.” (Participant 3, Clinical and Allied Health).

#### Divergent attitudes to post-treatment survivorship

There was the spectrum of attitudes toward post-treatment supportive care evident among the participants interviewed. While there was a general consensus that post-treatment supportive care is important, attitudes varied on how it should be prioritised within the cancer continuum. Some felt that post-treatment supportive care was vital, and should be incorporated into survivorship planning at the point of diagnosis:

“we should be planning for post-treatment from diagnosis. Obviously getting the biomedical stuff right is crucial, but we should also be thinking about and planning for survivorship from the beginning, rather than leaving them to work it out for themselves when things to begin to unravel.” (Participant 41, Primary Health Provider)

While participants at the other end of the spectrum acknowledged the value of post-treatment supportive care, but felt that given the resources and capacity currently available, it should not be prioritised, as Participant 10 (Clinical and Allied Health) exemplifies:

“As health professionals, we know that there’s obviously going to be that survivorship pathway after treatment, and I think it’s gaining momentum and traction and acceptance, but it seems a bit of a plus, you know, the icing on the cake, rather than vital. It’s not a priority focus. If we don’t get the treatment pathway right, there is no survivorship.”

#### Limited implementation of survivorship models of care

In addition to the lack of ownership or leadership in the survivorship space, there also appeared to be limited implementation of survivorship models of care in the provision of post-treatment supportive care. While a small number of participants did refer tangentially to a survivorship model of care, for example “our model of care is very survivorship focussed” (Participant 30, Supportive Care Provider), there was no reference to any specific survivorship models, nor any articulation of how they are being operationalised within the provision of supportive care for post-treatment cancer survivors.

#### Absence of survivorship care plans

Survivorship care plans are also markedly absent in the provision of post-treatment supportive care in NZ, with the comments of Participant 1 typifying the majority of responses:

“We haven’t got a survivorship plan for people. There’s been lots of discussion in the past that there should be survivorship plans, but we don’t use them” (Participant 1, Clinical and Allied Health).

While the quote above hints at an acknowledgement that survivorship care plans should be adopted as best practice, Participant 28 makes the point more explicitly:

“Survivorship care plans are considered the gold standard in Australia, and I think we should be doing that here” (Participant 28, Supportive Care Provider).

#### Lack of financial resources and capacity

Participants consistently cited a lack of financial resources and/or capacity as a significant barrier to the provision of post-treatment supportive care. Participant 5 highlights the limited public funding for services in NZ:

“So the biggest barrier is funding, there is little public money for post-treatment services. You know, I can talk to a patient, give them some basic advice, but there’s really nothing else I can offer them unless they can afford to pay for it.” (Participant 5, Clinical and Allied Health).

Whereas Participant 7 describes how the provision of post-treatment supportive care is severely restricted by a chronic lack of capacity in the healthcare workforce, a point embodied by Participant 1.

“I also think, health professionals, we think we do a good job most of the time. But we don’t always actually do what we say we’re going to do. When you have people, at the very breaking point of just being able to do the basics of their job, and keep people well and safe, physically. It’s really, really hard to do those additional layers of best practice care.” (Participant 7, Clinical and Allied Health)

“Please don’t make that [survivorship care plans] one of your recommendations, we are stretched so thin as it is.” (Participant 1, Clinical and Allied Health).

### Equity in supportive care provision for cancer survivors post-treatment

#### Provision of supportive care for underserved populations

As outlined earlier, few supportive care services and programmes specifically target the post-treatment needs of cancer survivors, so it is unsurprising that services and programmes specifically targeting the post-treatment needs of under-served populations are almost non-existent:

“This is what keeps me awake at night. What’s happening with Māori, Pacific and Asian folk who are going through a cancer experience, and how do they connect in to get the support services they need. And I just don’t see an adequate plan in place for those folk. I think they are hugely vulnerable, and if you look at the statistics, they don’t do well following treatment, but there doesn’t seem to be a plan in place.” (Participant 26, Supportive Care Provider).

When discussing services for under-served populations, participants tended to refer to broader health system services that provide cultural support, language translation, and transport. For example, services mentioned included: DHB-based Māori and Pacific support services, or cancer specific services situated in both DHBs and the community, such as Māori and Pacific cancer navigators, rather than services specifically targeting post-treatment needs.

#### Equity in cancer care more broadly

Equity in cancer care was considered important across the spectrum of participants, but largely framed in deficit discourse, as evident in the comments of Participant 4 (Clinical and Allied Health):

“We certainly need more Māori and Pacific cancer co-ordinators, because they are just invaluable. I cannot tell you how many patients that would not have engaged with us, had it not been for the Māori and Pacific cancer co-ordinators that we do have. They quite literally save lives.”

Despite acknowledging the importance of equity in cancer care in NZ, participants generally felt that it is not currently prioritised. The comments below of Participant 1 (Clinical and Allied Health) and Participant 2 (Māori Health Provider) illustrate this point, describing a mono-cultural system, ill-equipped to deal with the diverse ethnic population it serves:

“We are such a hospital-based system. We are a very hospitalised, very white service, traditionally” (Participant 3, Clinical and Allied Health).

“Our Māori whānau, I think a lot of them like to be connected with other Māori. And when you consider that actually most Māori go through the cancer journey and they don’t see one Māori face, you know, it can be terrifying. Imagine being a Pākehā going through treatment, and you never saw another Pākehā face – that’s kind of scary, eh.” (Participant 2, Māori Health Provider)

Barriers to equity being prioritised in cancer care commonly noted by participants included a lack of capacity; lack of accountability for failing to meet cultural competencies; and systemic racism, as the following comments elucidate:

“Equity factors around ethnicity, distance to treatment centre, health literacy, personal resources, economic factors still hugely impact cancer outcomes. But an expression that I use frequently is lack of capacity will always knock over equity.” (Participant 7, Clinical and Allied Health)

“We have a very mono-cultural service, that deals with some people really well, but not everybody, and we see the outcomes of it…We’ve got a whole lot of people in the healthcare workforce, who all have cultural competencies, or Te Tiriti o Waitangi competencies, but year in, year out, just seem to get signed off, with nothing tangible or accountable put in place.” (Participant 27, Māori Health Provider)

“Systemic racism is definitely a barrier, and then there is that whole clinical versus non-clinical thing. Health is a very hierarchical system, you know, and we’re (non-clinical Māori Health providers) right at the bottom.” (Participant 44, Māori Health Provider)

#### Geographical disparity in the provision of supportive care

Participants also indicated that large geographical variation exists in the provision of post-treatment supportive care:

“There is definitely a postcode lottery. What services you can connect into depends on where you live.” (Participant 32, Supportive Care Provider)

This geographical disparity generally manifests in two distinct ways. First, as Participant 34 highlights, some specialised services are only available in certain parts of the country:

“[our service] is just not out there, unless they are coming to us specifically. I mean, there is one other place here in Auckland, but that is it. So if you don’t live in Auckland, or can’t travel to Auckland, then you just can’t access [our service]” (Participant 34, Clinical and Allied Health).

And second, the range of services are prioritised differently in different parts of the country, even within the same organisation, as participant 24 explicates:

“We have several regions, and our national office, and a lot of expertise, but it’s kind of not all together, so everyone is often doing different things. That is something we could do better.” (Participant 24, Supportive Care Provider)

While the comments of Participant 24, above, frames this disparity negatively, many participants argued that some regional autonomy needs to be retained in order to meet the needs of specific populations, within a national base standard of care:

“There will always be nuances…we have a big rural population, so we really need to make sure the needs of rural people are met, but Auckland has a huge Māori/Pacifica population. So you have to say hey, we are not the same, there will be nuances that fit that geographical population disparity, but everyone can expect a base model of care. This is where I think, hopefully, this New Zealand Health reform is going to be better.” (Participant 32, Supportive Care Provider)

## Discussion

In line with international literature, the findings of this study of 47 healthcare providers involved in the treatment and supportive care of cancer survivors indicate that cancer survivors in NZ face a wide-range of psychosocial and physical issues following treatment. Psychosocial issues include a sense of abandonment; fear of recurrence; emotional and psychological processing; a sense of abnormality; returning to the workforce; and financial implications; while physical issues range from loss of strength through to loss of a body part. In 2006, the American Institute of Medicine published a seminal report entitled ‘From Cancer Patient to Cancer Survivor”, which outlined 10 recommendations aimed at improving the provision of care for cancer survivors to help address these unmet needs. One of the key high-level recommendations from this report was that healthcare providers, patient advocates, and other stakeholders should work to establish cancer survivorship as a distinct phase of cancer care, raise awareness of the needs of cancer survivors, and act to ensure the delivery of appropriate survivorship care [5]. There have been some advances in the cancer survivorship space in NZ in the ensuing years, notably the consensus statement on cancer survivorship in NZ, leading to greater emphasis on survivorship within national cancer planning [1, 25, 26]. But this study clearly highlights that more needs to be done in relation to implementing this recommendation in the NZ context. While healthcare professionals participating in this study, themselves, articulated a range of psychosocial and physical needs common among cancer survivors post-treatment in NZ, they also indicated a lack of awareness of such needs among the wider public and whānau members of cancer survivors, and feelings of abnormality associated with such needs among cancer survivors themselves. It was also clear that there are significant gaps in service provision explicitly targeting the post-treatment needs of cancer survivors in NZ, with solutions primarily sought within the existing fragmented and stretched supportive care services, rather than specific survivorship supportive care services.

One of the key barriers to the establishment of cancer survivorship as a distinct phase of cancer care in NZ, identified by this study, was the lack of ownership of the post-treatment survivorship space. The provision of post-treatment supportive care currently sits between treatment providers, primary care providers, and a suite of cancer NGOs and allied health providers in the community, with no overarching leadership or framework to guide the co-ordination of care for cancer survivors. International literature clearly highlights the benefits that each category of healthcare provider outlined above individually brings to survivorship care [34–39], but there is also evidence that their respective responsibilities are often poorly defined, and that their provision of survivorship care is generally sub-optimal beyond their area of speciality [2, 34, 39–46]. So while it is vital that the provision of long-term follow-up care in NZ remains multi-disciplinary, it is also imperative that the contributions of each discipline are guided in a more structured way.

The recently established *Te Aho o Te Kahu* (the Cancer Control Agency) in NZ, is perhaps best positioned to fill the leadership vacuum in survivorship care, given its wide-ranging purview to work with partners across the cancer continuum to improve cancer care outcomes for all New Zealanders [47]. At an operational level, the Cancer Society is well placed, given that it works across tumour streams, has a wide presence across NZ, and is already considered a key provider of post-treatment supportive care services. Nurse practitioners and cancer nurse coordinators could also play a significant leadership role in post-treatment survivorship care, given their strengths in education, navigation, and their engagement with patients throughout the cancer journey [19, 37, 48]. Given the current co-governance direction set by the Government, working in partnership with Māori would be critical to meet the needs and aspirations of tangata whenua. More broadly, the recent health reforms in NZ, notably the centralisation of governance and establishment of the Māori Health Authority, present potential opportunities for streamlining post-treatment supportive care in NZ.

The other high-level barrier to more structured post-treatment survivorship care evident in this study was the lack of development and/or implementation of a model, or model(s), of care. Many models of care for cancer survivorship have been researched and developed in the past decade, for example risk stratification models, chronic care models, and transition models [22, 23], but there is little evidence of the efficacy of these models [2, 22–24]. While it is beyond the purview of this study to describe the myriad of cancer survivorship models of care that have been developed in any great depth, the current absence of survivorship models identified by this study indicates the need for greater identification and/or development, and implementation of survivorship models of care, in the provision of post-treatment supportive care in NZ. It is generally accepted that there is currently no optimal model of care that will work for all cancer survivors [2, 23, 24], but a broad, holistic, integrated model that can be tailored to address individual needs, could help clarify practitioner roles and responsibilities, and ensure more equitable access to post-treatment survivorship care [24].

Survivorship care plans are also notably absent from the post-treatment supportive care landscape in NZ. A survivorship care plan is a written record of a cancer survivor’s diagnosis, treatment, and recommended follow-up care, advice on maintaining their health and well-being and monitoring for recurrence, and information about the availability of psychological and social support [49–51]. Providing all cancer patients with a survivorship care plan was another key recommendations made in the American Institute of Medicine report in 2006 [52], and they have since been adopted as best practice in a number of countries [11, 12, 53]. Some studies have shown that cancer survivors and healthcare providers find supportive care plans beneficial to co-ordination of care, improved referral pathways, the delineation of roles and responsibilities, and provision of information on follow-up care [14–17]. However, there is considerable variation in the design and implementation of survivorship care plans [13, 14], and a paucity of evidence of survivorship care plans measurably improving cancer survivor’s health outcomes [2, 16, 20]. Further research is needed to test different variations of survivorship care plans, and their efficacy and cultural safety, in a range of settings [17, 20, 21].

There is also a growing literature describing the barriers to the provision of survivorship care plans to cancer survivors. Key barriers to survivorship care plan use include the time needed for preparation of the plans; the lack clarity around who is responsible for preparing the plan and lack of reimbursement for this role; poor communication and collaboration between providers; and the lack of data on the association between the use of survivorship care plans and patient outcomes noted above [2, 16, 18, 19]. These barriers are concordant with the findings in this study which highlight limited capacity and financial resources as key barriers to the provision of post-treatment supportive care in NZ.

There are some limitations to our findings. First, participants were drawn from across the country which, while providing a good overview of the post-treatment supportive care landscape in NZ, made it difficult to capture the nuances of service provision at the local level, or make meaningful comparisons between different areas. Second, the sample was imbalanced between the different categories of healthcare providers, with fewer participants coming from primary health providers and Māori health providers than planned. As noted above, international literature indicates that different categories of healthcare providers bring different strengths and biases to post-treatment survivorship care, so this imbalance in the sample may have skewed our findings. Moreover, while the sample included participants working in both tumour-specific and cross-tumour settings, not all tumour-specific streams were represented. As different tumour types require different treatments, there is wide variation in the post-treatment needs of cancer survivors, and support services required to meet them, which may not have been exhaustively captured here. Third, participants were almost exclusively female and predominantly Pākehā. While this is, in some ways, reflective of the supportive care and cancer nurse workforce in NZ [54], the lack of gender and ethnic diversity in the sample is a limitation of this study. And finally, while the exclusion of the voice of cancer survivors could also be considered a limitation of this study, a study exploring the issues faced by cancer survivors post-treatment in NZ from their own perspective was undertaken concurrently, and will be reported on separately.

In summary, cancer survivors in NZ face a range of psycho-social and physical issues post-treatment that require supportive care. At present, the provision of supportive care to meet these needs is fragmented and inequitable. In order to raise awareness of the issues faced by cancer survivors, and improve the provision of post-treatment supportive care in NZ, cancer survivorship needs to be established as a distinct phase of cancer care, in line with the recommendation of the American Institute of Medicine. Measures to help achieve this could include greater leadership in the survivorship space; the development and/or implementation of a survivorship model(s) of care; and the use of survivorship care plans; all of which could help improve referral pathways, and clarify clinical responsibility for survivorship care. The key barriers to implementing such measures include a lack of capacity and resources within the existing cancer care framework; divergent attitudes to survivorship care within the cancer care workforce; and a lack of clarity around whose responsibility post-treatment survivorship care is. Future research could include kaupapa Māori approaches, the development, implementation and evaluation of model(s) of care, survivorship care plans, and/or programmes and services specifically targeting the post-treatment needs of cancer survivors. Future research could also explore ways in which the contributions of different types of healthcare providers could be guided in a more structured way to help improve referral pathways, and clarify clinical responsibility for survivorship care.

## Data Availability

The datasets generated and/or analysed during this study are not publicly available, but are available from the corresponding author on reasonable request.
